# „Digitale Einzelhandelsplattformen und städtische Akteur*innen – Kooperation als zukunftsfähiges Modell?“

**DOI:** 10.1007/s00548-023-00845-2

**Published:** 2023-03-21

**Authors:** Sina Hardaker, Alexandra Appel, Paulina Doll, Kerstin Ströbel

**Affiliations:** 1 Institut für Geographie, Lehrstuhl für Wirtschaftsgeographie, JMU Würzburg, Am Hubland, 97074 Würzburg, Deutschland; 2 IKEM – Institut für Klimaschutz, Energie und Mobilität, Magazinstr. 15–16, 10179 Berlin, Deutschland

**Keywords:** Stationärer Einzelhandel, Innenstadt, eBay, Plattformökonomie, Lokalität, Brick-and-mortar retail, City centers, eBay, Platform economy, Locality

## Abstract

Vielerorts werden lokale Onlinemarktplätze ins Lebens gerufen, um innerstädtische Einzelhändler*innen vor dem Hintergrund des Strukturwandels, der coronabedingt verstärkt wurde, digital besser aufzustellen. Anhand eBay Deine Stadt, einer Initiative für lokale Onlinemarktplätze von eBay, die seit 2020 in Deutschland in bislang über 30 Städten und Regionen etabliert wurde, beleuchtet der Artikel die Strukturen und Akteur*innen, die an der Umsetzung der Initiative beteiligt sind. Auf Grundlage von Expert*inneninterviews mit einem von eBay für die Umsetzung beauftragten Beratungsunternehmen sowie kommunalen Akteur*innen werden erste Erfahrungen, Erwartungen und Kritikpunkte erhoben und diskutiert. Ziel ist es, eine anfängliche Evaluation des sich noch im Aufbau befindlichen lokalen Onlinemarktplatz-Programms eBay Deine Stadt durchzuführen. Dabei stehen insbesondere die Rolle eBays als Infrastrukturgeber und Kooperationspartner sowie die damit einhergehenden Prozesse, Probleme und Erwartungen im Vordergrund. Darauf basierend werden weiterführende Fragestellungen zu zukünftigen Dynamiken von Innenstädten identifiziert.

## Abstract (en)

Local online marketplaces are being launched in many cities in order to better digitally position inner-city retailers against the backdrop of structural change. This article sheds light on the structures and actors involved in the implementation of eBay Deine Stadt, an initiative for local online marketplaces by eBay that has been established in more than 30 cities and regions in Germany since 2020. Based on expert interviews with a consulting firm commissioned by eBay for the implementation, as well as with municipal actors, the initial experiences, expectations, and criticisms are discussed. The goal was to conduct an initial evaluation of the local online marketplace program eBay Deine Stadt. The article thereby focuses on the role of eBay as an infrastructure provider and cooperation partner, as well as the associated processes, problems, and expectations. Based on the findings, further questions concerning future dynamics of city centres will be identified.

## (Lokale) Onlineplattformen für Einzelhandel und Innenstadt

Vielerorts setz(t)en sich städtische Akteur*innen mit lokalen Onlineplattformen auseinander, um den stationären Einzelhandel in (Innen‑)Städten zu unterstützen und eine virtuelle Erreichbarkeit zu gewährleisten. Vor diesem Hintergrund werden lokale Onlinemarktplätze als Plattformen verstanden, „deren Angebot einem begrenzten geographischen Raum (z. B. Stadt oder Region) zugeordnet werden kann und deren Fokus auf der lokalen Wertschöpfung sowie der Sichtbarkeit der einzelnen Akteur*innen, insbesondere Händler*innen liegt“ (Hardaker 2022a, S. 171). Erste Studien zeigen, dass E‑Commerce- und Onlinemarketing-Strategien die Resilienz stationärer Händler*innen in Zeiten der coronabedingten Lockdowns, aber auch hinsichtlich des Strukturwandels und der weiterhin bestehenden Herausforderungen aufgrund Konsumzurückhaltung und Energiekrise maßgeblich begünstig(t)en (Appel und Hardaker 2021; Hardaker et al. 2022). Im Zuge dessen haben sich unterschiedliche Modelle lokaler Onlineplattformen entwickelt, die jedoch größtenteils kritisch und als wenig erfolgreich eingeschätzt werden (Battermann und Neiberger 2018; Hardaker 2022a). Argumentiert wird i. d. R. mit der fehlenden Reichweite lokaler Plattformen aufgrund ihres lokalen Charakters und des dadurch eingeschränkten Angebots. Unabhängig von der kategorischen Einteilung von Plattformen haben diese gemeinsam, dass sie von Netzwerkeffekten (z. B. Kenney und Zysman 2016; Srnicek 2017) profitieren: Dies bedeutet, dass die Attraktivität von Plattformen mit ihrer Netzwerkgröße wächst, d. h. je mehr Teilnehmer*innen (z. B. in Form von Händler*innen und Kund*innen) die Plattform nutzen, desto mehr weitere Nutzer*innen zieht sie an. Somit werden digitale Plattformen als mehrseitige Vermittler verstanden, die es unterschiedlichen Akteur*innen ermöglichen, zueinander zu finden und sich zu vernetzen (*matchmaking*) (Evans und Schmalensee 2016).

Der wachsende Einfluss und die zunehmende Präsenz digitaler Plattformen in Städten führen zur Etablierung eines Plattformurbanismus (z. B. Hardaker 2021; Sadowski 2020), der dadurch geprägt ist, dass Unternehmen wie Google, Uber oder Amazon gesellschaftliche Strukturen und Räume (re-)produzieren und an der städtischen Organisation und den Dynamiken teilnehmen. Über die Bereitstellung digitaler Plattformen (re-)organisieren und steuern sie eine Vielzahl städtischer Interaktionen und Abläufe zwischen Nutzer*innen, Arbeitnehmer*innen, Kapital und Informationen in Bereichen wie Verkehr, Wohnen und Einkaufen (Grabher and van Tuijl 2020; Richardson 2020).

Stationäre Händler*innen scheinen mehr und mehr in den Fokus etablierter Plattformen zu treten (Hardaker 2022b). Dies trifft auch auf die 1995 in Kalifornien gegründete Handelsplattform eBay zu. In Deutschland möchte eBay mittels externem Beratungsunternehmen mit Städten und Regionen kooperieren und insbesondere (inner-)städtische Einzelhändler*innen für die von eBay initiierten, als lokal deklarierten Marktplätze eBay Deine Stadt gewinnen. Damit stellt eBay Deine Stadt ein Format dar, mit welchem Händler*innenzahlen in Zeiten starken Wettbewerbs mit anderen digitalen Plattformen, u. a. Amazon, erhöht werden sollen.

Der vorliegende Beitrag untersucht die Strukturen von eBay und kommunalen Akteur*innen während der Etablierung von eBay Deine Stadt in Deutschland und beleuchtet wie eBay im Rahmen dieser Kooperationen kommunale und gesellschaftliche (Infra‑)Strukturen mitgestaltet. Insgesamt wurden 20 Expert*inneninterviews zwischen März 2022 und August 2022 mit dem Beratungsunternehmen sowie kommunalen Akteur*innen durchgeführt und erste Erfahrungen, Erwartungen und Kritikpunkte erhoben und diskutiert. Damit leistet die Arbeit einen Beitrag zu einem besseren Verständnis der Rolle digitaler Plattformen und deren Auswirkungen auf lokale und kommunale Strukturen sowie zur Diskussion über die Lokalisierbarkeit digitaler Verkaufsformate.

## eBay Deine Stadt

**Abb. 1:**
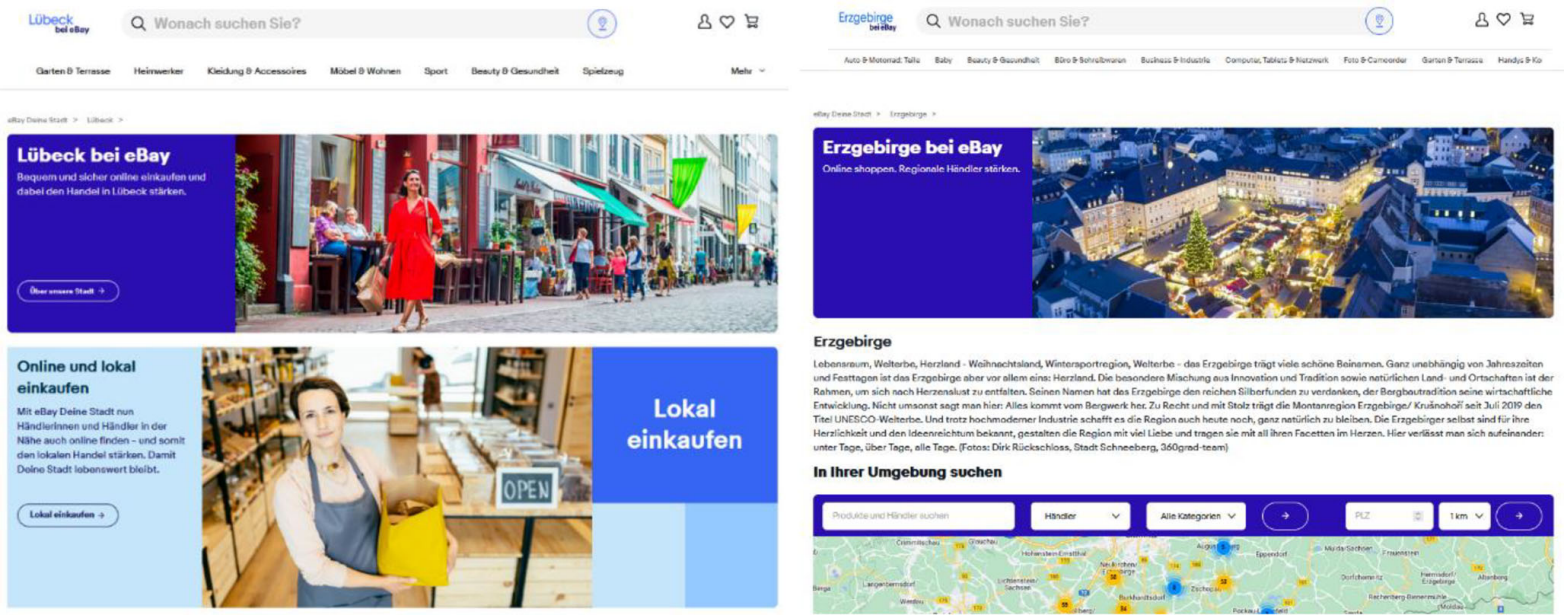
eBay Deine Stadt Lübeck und Erzgebirge. Städte und Regionen haben die Möglichkeit, sich auf der Website vorzustellen. (Screenshots, eBay 2022)

Laut eBay Deutschland ist eBay Deine Stadt eine „bundesweite Initiative …, die es Städten und Kommunen [ermöglicht], lokale Online-Marktplätze einzurichten“ (eBay 2021) und 2020 in Kooperation mit dem Deutschen Handelsverband (HDE) etabliert wurde. Das Besondere an der Initiative ist, dass die Händler*innen mit ihren Produkten nicht nur auf den lokalen Plattformen, sondern zeitgleich auf dem nationalen eBay-Marktplatz vertreten sind. eBay Deutschland argumentiert, dass durch die Einbettung in die nationale eBay-Plattform bereits zum Start der lokalen Plattform eine kritische Masse an Händler*innen und Angeboten aus der jeweiligen Region vorhanden ist, da alle bereits über eBay Handelnde in das lokale Profil integriert werden. Auf den jeweiligen Homepages werden lokale Besonderheiten hervorgehoben – in Würzburg etwa lautet der Slogan „Einfach fränggisch. Online shoppen in der Domstadt“ – und Bildmotive aus der jeweiligen Innenstadt sowie ausgewählte Einzelhändler*innen dar- bzw. vorgestellt (eBay 2021; Abb. 1).

**Abb. 2:**
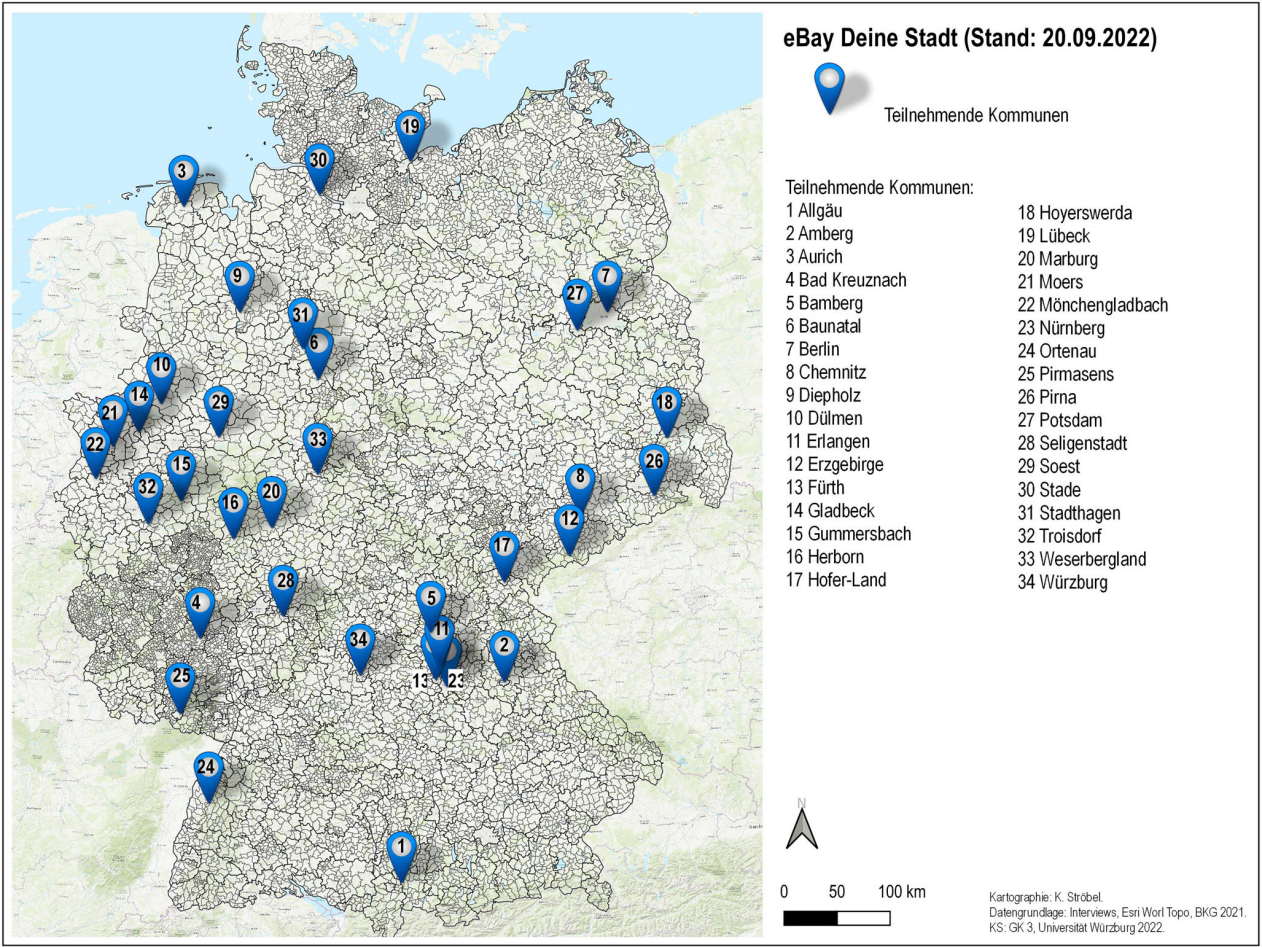
Teilnehmende Städte, Landkreise bzw. Regionen eBay Deine Stadt. (Quelle: eigene Darstellung auf Grundlage der eBay Deine Stadt Homepage)

Während die Initiative im Jahr 2020 mit zehn Städten startete, sind derzeit 34 deutsche Städte und Regionen laut eBay Deutschland mit ca. 15.000 Händler*innen und einem Sortiment von ca. 30 Mio. Artikeln auf dem Onlinemarktplatz vertreten (Abb. 2). Laut Werbevideo besitzen rund die Hälfte der eBay Händler*innen ein stationäres Ladengeschäft (eBay 2022). Alle Interviewpartner*innen bestätigen, dass bisherige Versuche, Onlinemarktplätze zu etablieren, weitestgehend als gescheitert angesehen werden können. eBay Deine Stadt wird vielerorts als derzeit realistische Möglichkeit zur Etablierung eines lokalen Onlinemarktplatzes angesehen, u. a. wegen der bereits vorhandenen Infrastruktur, kritischen Masse und Integrationen in eine Plattform mit translokaler Reichweite, die potenziell gewünschte Netzwerkeffekte mit sich bringt. Damit hebt sich eBay entscheidend von bislang entwickelten lokalen Onlinemarktplätzen ab:

„Es gibt für mich keinen regionalen Onlinemarkt der wirklich funktioniert und der einzige Ansatz regionale Onlinemärkte zu machen, die funktionieren können, ist meiner Meinung nach, wenn man versucht mit großen Plattformen mit Reichweite zusammenzuarbeiten. Das andere ist ein Stück kommunale Selbstbefriedigung … und eBay war das Erste, was halbwegs überzeugt hat“ (Interview V).

## eBay als Infrastrukturgeber und Kooperationspartner

**Abb. 3:**
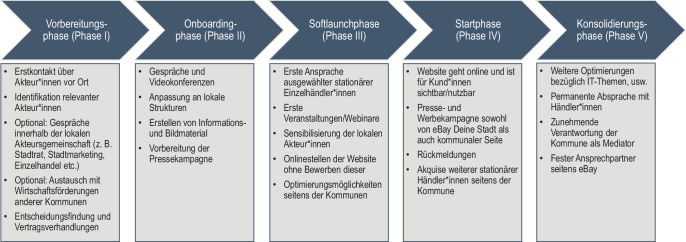
Schematische Darstellung des Etablierungsprozesses (Grafik: Kerstin Ströbel, eigene Darstellung auf Grundlage der Expert*inneninterviews)

Auf Grundlage der geführten Interviews kann die Etablierung von eBay Deine Stadt in verschiedene Phasen eingeteilt werden. Die Identifikation der in den unterschiedlichen Phasen involvierten Akteur*innen und Arbeitsschritte lässt Rückschlüsse über den Arbeits- und Zeitaufwand der Beteiligten zu – ein Aspekt, der sowohl für Kommunen als auch für Einzelhändler*innen als große Herausforderungen bei der Etablierung von lokalen Onlinemarktplätzen gesehen wird (Interview V). Der Etablierungsprozess lässt sich schematisch in fünf Phasen untergliedern (Abb. 3).

Charakteristisch für die Vorbereitungsphase (Phase I) ist die Kontaktanbahnung zwischen dem externen Vertreter von eBay Deutschland und den jeweiligen Kommunen/Regionen. Der Großteil der interviewten Kommunen berichtet, dass der Erstkontakt auf Initiative von eBay über E‑Mail oder telefonisch erfolgte. Im Namen von eBay Deutschland kontaktierte das Beratungsunternehmen Vertreter*innen der städtischen Wirtschaftsförderungen und/oder private Stadtmarketinginitiativen (Interview XV). In einem Fall ergriff eine langjährige eBay-Händlerin die Initiative und machte die lokale Wirtschaftsförderung auf das Projekt aufmerksam (Interview X). In Phase I spielen zudem die Identifikation verantwortlicher Akteur*innen auf kommunaler Seite und deren Entscheidungsfindung eine wichtige Rolle (Interview V; XIX; XVI).

Die Kommunen/Regionen verfolgen im Rahmen der Vorbereitung und Entscheidungsfindung sehr unterschiedliche Strategien. Während eine Stadt von einer Stärken-Schwächen-Analyse der Innenstadt berichtet, hat eine andere einen Maßnahmenplan vorgelegt, der über den Onlinemarktplatz inhaltlich hinausgeht und in den Stadtrat eingebracht wurde. Andere wiederum holten Meinungen der Handelsverbände, Stadträte und Senatsverwaltungen sowie der örtlichen Industrie- und Handelskammern ein (Interview I, XIII, XIV, XV, XIX). Wieder andere befragten Einzelhändler*innen (Interview XV) und/oder Vertreter*innen der Wirtschaftsförderung bzw. des Stadtmarketings anderer Standorte (Interview V). Die Kooperation mit dem HDE kann als Multiplikator gesehen werden, wie das folgende Interviewzitat zeigt (Interview V): „Dann haben wir mitbekommen, dass der hessische Handelsverband das empfiehlt und … haben dann mit dem Handelsverband gesprochen.“

In Phase II werden Kooperationsstrukturen aufgebaut und eBay Deutschland geht gemeinsam mit der Kommune/Region in die Onboarding-Phase der Homepage über, in der Backend-Funktionen und IT-Auftritte von eBay Deine Stadt angepasst und die lokale Online-Repräsentation der Stadt in die nationale eBay Deutschland-Plattform sowie die eBay Deine Stadt-Homepage integriert werden. Charakteristisch ist zudem die Vorbereitung der Pressekampagne (Interview V). Einzelne Städte fertigen gezielt Fotos mit professionellen Fotograf*innen für die eBay Deine Stadt-Website an (Interview I). Ein Interviewpartner verweist auf eine Vielzahl von Videokonferenzen und Telefongesprächen zwischen Kommunen und eBay. Im Fokus des Austauschs stehen u. a. die inhaltliche und (foto-)grafische Aufbereitung der lokalen Marktplatzhomepages, was den damit verbundenen zeitlichen und personellen Aufwand von kommunaler Seite verdeutlicht (Interview XIII). Dies widerspricht dem von eBay suggerierten Bild einer „Plug & Play“-Installation von eBay Deine Stadt. In dieser Phase finden Anpassungsprozesse an lokale Strukturen und Wissenstransfer statt. Beispielsweise war die ursprüngliche Idee von eBay, sich ausschließlich an Städte zu wenden, was sich im Laufe der Zeit geändert hat. Das Beispiel von Regionen verdeutlicht den Lernprozess:

„Die Landkreise hatten wir gar nicht so auf dem Zettel. … Weil dann einfach so gewisse kleinere Städte gebündelt sind und das dann wieder einfacher in der kommunalen Organisation ist als auch sich in der Nutzerwahrnehmung ja schon irgendwie besser abbilden lässt“ (Interview III).

Die Interviewpartner*innen der Kommunen/Regionen berichten von höchst unterschiedlichen Lösungen für die personellen Verantwortlichkeiten – teils werden neue (Teil‑)Stellen geschaffen, teils werden verantwortliche Kümmerer eingesetzt oder zusätzliche Arbeitspakete entstehen für die bereits involvierten Mitarbeitenden.

In Phase III folgt das Onlinestellen der Website, jedoch wird diese noch nicht kundenseitig beworben. Im Mittelpunkt stehen die technische Optimierungen, teils händlerseitige Aufbereitung von Repräsentationsmaterial für den Onlineshop und das Vertrautmachen der Kommunen mit dem System. Teilweise werden die Einzelhändler*innen in Phase III eingebunden, um auch ihre Meinungen einbringen zu können. Ein Interviewpartner (V) verweist auf den Austausch mit den Händler*innen: „Wir haben Erstgespräche auch mit Händlern geführt, auch Seminare durchgeführt und die Leute dafür vorab sensibilisiert.“ Mit eBay zusammen werden Webinare veranstaltet und Informationen an Einzelhändler*innen verteilt (Interview I; XVI).

In Phase IV geht der lokale digitale Marktplatz mit den bereits vorhandenen eBay-Händler*innen der Kommune/Region online und die kundenseitige Öffentlichkeitsarbeit dominiert. Die Kommunen akquirieren – teilweise sehr aufwendig – zusätzliche Händler*innen (z. B. Interview V, und viele mehr). In einigen Regionen werden vonseiten der Wirtschaftsförderung auch Besuche vor Ort getätigt.

In der Konsolidierungsphase (V) stehen Kommunikationsprozesse zwischen Kommune/Region und eBay sowie zwischen Kommune/Region und Händler*innen im Fokus, wobei die/der von kommunaler Seite eingesetzte Verantwortliche zunehmend als Mediator fungiert (Interview III). Parallel finden Optimierungsschritte auf Basis der Rückmeldungen statt. Einige Interviews machen deutlich, dass es mit fortschreitender Laufzeit der Initiative und der einhergehenden steigenden Anzahl an Kommunen zu Verzögerungen kommt:

„Das Problem ist halt bei solchen Projekten, je größer, je mehr Köche, da geht es nicht mehr so schnell mit einzelnen kleinen Umsetzungswünschen“ (Interview XV).

Dabei ist die permanente Absprache zwischen Kümmerer und Einzelhändler*innen vor Ort von maßgeblicher Bedeutung. Dies wird nur in einzelnen Fällen durch das eBay Durchstarterprogramm abgefangen, welches gewerblichen Händler*innen, die sich kürzlich bei eBay registriert haben, unter bestimmten Voraussetzungen zur Verfügung steht. Von nun an erhält die teilnehmende Kommune/Region quartalsweise Berichte von eBay, die u. a. die Anzahl der teilnehmenden Einzelhändler*innen je Standort darlegen (Interview VII). Die Phase ist zudem geprägt von Überzeugungsarbeit gegenüber potenziellen Händler*innen: „man trifft sich, man konsolidiert noch, wir suchen noch nach weiteren Händlern“ (Interview V).

## Erste Bewertungen von eBay Deine Stadt durch kommunale Akteur*innen

eBay wird von den meisten Kommunen als Partner angesehen, der ansprechbar ist und aufgrund des externen Beratungsunternehmens eine gewisse Zugänglichkeit aufweist. Für viele scheint eBay die einzige realistische Chance für lokale Onlinemarktplätze darzustellen. Insbesondere die ersten zehn teilnehmenden Kommunen hatten vermehrt Möglichkeiten der Mitgestaltung, was als positiv wahrgenommen wurde (Interview IX, XIX). Die meisten Interviewpartner*innen berichten jedoch von einem mehrheitlich fehlenden Interesse der Händler*innen, bei eBay Deine Stadt mitzuwirken. Zudem werden teils starke Vorbehalte gegenüber eBay erwähnt. „Mit denen wollen wir nichts zu tun haben“ und „Schnappatmung … beim Wort … eBay“ unterstreichen die teilweise ablehnende Haltung der Einzelhändler*innen gegenüber dem Projekt (Interview XV). Der Ablauf sei durch viel Frustration über einen stellenweise destruktiven Austausch geprägt, durch den viel Zeit „verschwendet“ (Interview XIX) wurde. Dies, kombiniert mit technischen Problemen, wurde als Hauptgrund für gescheiterte Kooperationen angeführt. In zwei Fällen steht bereits fest, dass die Verträge nicht verlängert werden und sich die Städte wieder vom Marktplatz verabschieden. Zudem zieht sich durch viele Städte der Kritikpunkt, dass die Ressourcen fehlen, um eBay Deine Stadt längerfristig zufriedenstellend bedienen zu können in Form eines Kümmerers (ebd.). Dem gegenüber stehen Interviewpartner*innen, die von Händler*innen berichten, die problemlos an den Marktplatz angebunden werden und bereits Umsätze generieren konnten (Interview IX).

Die in den von eBay in den Berichten genannten Informationen hinsichtlich Händler*innen und Produkten werden von den Kommunen teils unterschiedlich gedeutet. Während ein kleiner Teil der Befragten die Zahlen als positiv bewertet, verweist eine andere Gruppe auf eine unzulängliche Differenzierung der Berichte, die keine Rückschlüsse auf eine tatsächlich lokal stattfindende Entwicklung erlauben. So bleibt unklar wie viele der angegebenen Händler*innen eine stationäre Präsenz haben und wie sich die Umsätze in etwa verteilen. Erste Untersuchungen^1^ in ausgewählten Städten zeigen, dass lediglich 20–30 % der eingetragenen Händler*innen über ein stationäres Geschäft in der Innenstadt verfügen.

## Diskussion und Fazit

Mit eBay Deine Stadt hat ein weiterer Akteur die stationären (inhabergeführten) innerstädtischen Händler*innen in den Fokus genommen. Die interviewten städtischen Akteur*innen wurden dabei fast ausnahmslos von eBay direkt kontaktiert und hinsichtlich einer Kooperation angefragt. Dabei fällt auf, dass die Zuständigkeiten sehr unterschiedlich organisiert sind und es bislang kein standardisiertes Prozedere gibt. Generell zu erkennen ist der übergreifende Mix aus städtischen und privaten Akteur*innenbeteiligungen aus den Bereichen des Regionalmarketings und der Wirtschaftsförderung während des Etablierungsprozesses. Mehrere Städte betrachten ihren zunächst auf ein Jahr befristeten Vertrag eher als eine Art Testphase, um eBay Deine Stadt kennenzulernen. Dies bedeutet jedoch auch, dass seitens der städtischen Akteur*innen keine langfristigen Strategien bzgl. Zugangsrechte an erhobenen Daten, Abhängigkeiten von Plattformbetreiber*innen und ggf. einer kritischen Auseinandersetzung hinsichtlich privater Unternehmenskooperationen existieren.

Der Wille der Städte/Regionen, an eBay Deine Stadt teilzunehmen, war in vielen Fällen vor allem der Covid-19-Pandemie sowie fehlenden Alternativen und bereits negativen Erfahrungen mit gescheiterten lokalen Onlinemarktplätzen geschuldet. Laut mehreren Interviews (Interview I) „kam eBay wie gerufen“.

Es kann gezeigt werden, dass eBay Deine Stadt von kommunaler Seite häufig als Kooperationspartner zur Stärkung und Digitalisierung des stationären Einzelhandels und die bereits bestehende Plattform und Marke eBay als Mehrwert und aktuell als einzige realistische Chance für die Etablierung eines lokalen Onlinemarktplatzes gesehen werden. Bereits vorhandene eBay-Händler*innen, der HDE sowie ein persönlicher Ansprechpartner seitens des externen Beratungsunternehmens (eBay wird greifbar und bekommt ein Gesicht) führen dazu, dass eBay von einem Befürworter*innennetzwerk auf lokaler Ebene profitiert.

Interessanterweise spielt die Lokalität bei eBay jedoch nur während bestimmter Phasen (z. B. bei der Etablierung, Überzeugung lokaler Akteur*innen) eine Rolle. Später gerät der lokale Charakter letztlich in den Hintergrund, da die Händler*innen dann über eBay und damit auch ohne lokale Spezifikation auftreten bzw. aufgefunden werden können. Die lokale Einbettung kommt zu kurz, wie am Beispiel der fehlenden Integration lokaler Lieferdienste argumentiert werden kann, was einen weiteren Anreiz für „lokales Handeln“ darstellen könnte (Appel und Hardaker 2022). Letztlich verschwimmen bei eBay Deine Stadt die Grenzen zwischen lokalem Angebot und translokaler digitaler Sichtbarkeit.

Inwiefern die lokale Bewertung von eBay Deine Stadt als (nicht) erfolgreich ausfällt, scheint von stark variierenden Erwartungshaltungen und Bewertungskriterien der kommunalen Akteur*innen sowie der Onlineaffinität der stationären Händler*innen abhängig. Für viele ist zum Zeitpunkt des Interviews noch nicht abschätzbar, ob und wie erfolgreich der Onlinemarktplatz operieren wird. Zielvorgaben wurden in der Regel nicht konkret festgelegt. Fast allen teils sehr differenzierten Einschätzungen liegt nach wie vor ein Hauptproblem zugrunde: die Händler*innen zu mobilisieren. Im Rahmen dessen werden städtische Akteur*innen zu Akquisiteuren für eBay, indem sie stationäre Händler*innen – teils mit persönlicher Ansprache – von den Vorteilen des Onlinehandels überzeugen. Damit befördern städtische Akteur*innen eine positive lokale Bewertung und tragen potenziell auch zu einer gesamtgesellschaftlichen Legitimation der Etablierung solcher soziotechnischen Systeme wie Onlinehandel und Plattformökonomie/-urbanismus bei.

## Footnotes

1 Hildenbrand Y (unveröffentlicht, 2022) „eBay Deine Stadt“ – ein Projekt zur Unterstützung des stationären Einzelhandels. Eine Analyse der eBay Onlinemarktplätze von Amberg, Bamberg und Würzburg. Bachelorthesis. Julius-Maximilians-Universität Würzburg.
